# Genome-Wide Analysis of Single Nucleotide Polymorphisms Uncovers Population Structure in Northern Europe

**DOI:** 10.1371/journal.pone.0003519

**Published:** 2008-10-24

**Authors:** Elina Salmela, Tuuli Lappalainen, Ingegerd Fransson, Peter M. Andersen, Karin Dahlman-Wright, Andreas Fiebig, Pertti Sistonen, Marja-Liisa Savontaus, Stefan Schreiber, Juha Kere, Päivi Lahermo

**Affiliations:** 1 Department of Medical Genetics, University of Helsinki, Helsinki, Finland; 2 Finnish Genome Center, Institute for Molecular Medicine Finland, University of Helsinki, Helsinki, Finland; 3 Department of Biosciences and Nutrition, Karolinska Institutet, and Clinical Research Centre, Karolinska University Hospital, Huddinge, Sweden; 4 Department of Neurology, Umeå University Hospital, University of Umeå, Umeå, Sweden; 5 Popgen Biobank, Institute for Clinical Molecular Biology, Christian-Albrechts-University, Kiel, Germany; 6 Finnish Red Cross Blood Transfusion Center, Helsinki, Finland; 7 Department of Medical Genetics, University of Turku, Turku, Finland; 8 Department of General Internal Medicine, Institute for Clinical Molecular Biology, Christian-Albrechts-University, Kiel, Germany; 9 Folkhälsan Institute of Genetics, Biomedicum Helsinki, Helsinki, Finland; University of Otago, New Zealand

## Abstract

**Background:**

Genome-wide data provide a powerful tool for inferring patterns of genetic variation and structure of human populations.

**Principal Findings:**

In this study, we analysed almost 250,000 SNPs from a total of 945 samples from Eastern and Western Finland, Sweden, Northern Germany and Great Britain complemented with HapMap data. Small but statistically significant differences were observed between the European populations (F_ST_ = 0.0040, p<10^−4^), also between Eastern and Western Finland (F_ST_ = 0.0032, p<10^−3^). The latter indicated the existence of a relatively strong autosomal substructure within the country, similar to that observed earlier with smaller numbers of markers. The Germans and British were less differentiated than the Swedes, Western Finns and especially the Eastern Finns who also showed other signs of genetic drift. This is likely caused by the later founding of the northern populations, together with subsequent founder and bottleneck effects, and a smaller population size. Furthermore, our data suggest a small eastern contribution among the Finns, consistent with the historical and linguistic background of the population.

**Significance:**

Our results warn against *a priori* assumptions of homogeneity among Finns and other seemingly isolated populations. Thus, in association studies in such populations, additional caution for population structure may be necessary. Our results illustrate that population history is often important for patterns of genetic variation, and that the analysis of hundreds of thousands of SNPs provides high resolution also for population genetics.

## Introduction

Emerging genome-wide data are a powerful resource for analysis of population genetic variation, including population history and structure. These studies are of importance not only for researchers with historical interests, but also as a baseline for population-based studies of human disease, most notably association analyses of complex diseases where unknown population structure may introduce bias [1], [2]. Compared to previous methodology of human population genetics, the analysis of hundreds of thousands of loci across the genome allows a whole new level of accuracy and power without the constraint of having to use only a few loci as a proxy for the whole genome. This has already been demonstrated by a number of studies [e.g. 3]–[11].

We employed genome-wide SNP data to characterize genetic variation in Finland and Sweden in comparison with two reference populations from Germany and Great Britain, which have a Central European background and are larger, older and more admixed. Additionally, we also compared these data to the three HapMap populations from Europe, Africa and Asia [12].

The population history of Northern Europe has been reviewed earlier by several authors [13]–[20]. The settlement of the Baltic Sea region advanced rapidly after the Ice Age, beginning about 14,000 BC in Northern Germany and 10,000 BC in Finland. All the populations have their roots mainly in Central Europe, although some eastern influence has been observed among the Finns [21]–[23]. The early settlement in Finland covered almost exclusively the coastal and southwestern regions until a major settlement wave starting from central eastern Finland (the province of South Savo) led to the settlement of northern and eastern Finland from the 16^th^ century onwards. Even then, the population size throughout the country remained small, causing extensive genetic drift which, together with local and regional founder and bottleneck effects, led to the characteristic features of historical settlement of Finland: heavily drifted and isolated small breeding units. The results of this process have been seen in both common and especially rare autosomal alleles [13], [17]. Y-chromosomal studies have shown a strong genetic borderline between Western Finland and Eastern Finland [23]–[25], also supported by some studies of autosomal variation [26], [27]. Several studies have shown a longer range of linkage disequilibrium among the Finns, especially among the late settlement population of Eastern Finland, compared to the more outbred European populations [28]–[30].

Genetic variation in Sweden, Germany and Great Britain has been characterized less extensively than in Finland, and there is little evidence of strong population structure. In Sweden, mitochondrial DNA and Y-chromosomal studies indicate some geographical gradients [31], [32], and a pattern of local isolation has also been observed in northern parts of the country [33]; linkage disequilibrium studies indicate a lower extent of LD than among the Finns [34]. In Germany, only a minor degree of population structure between the northern and southern parts of the country has been detected by studies of autosomal markers [35], and some local differences by Y-chromosomal analysis [36]. Additionally, the German province of Schleswig-Holstein analyzed in this study has Y-chromosomal evidence [36] as well as historical records [37] indicating substantial admixture with the Danes. Genome-wide analysis of the British population has indicated only a slight genetic gradient from Southeast to Northwest, and the lack of strong substructure has been considered to be consistent with the multiple migrations that have affected the population [4].

**Figure 1:**
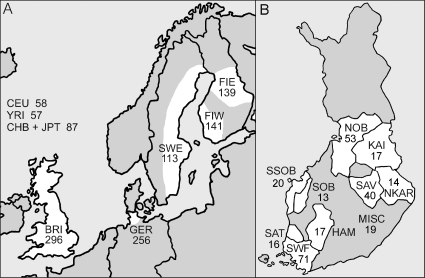
The map of Northern Europe (a) and Finland (b), and the sample sizes. The studied (sub)populations and their geographical ranges are shown in white. Abbreviations for the populations: Western Finland (FIW); Eastern Finland (FIE); Sweden (SWE); Germany (GER); Great Britain (BRI); Utah residents with ancestry from northern and western Europe (CEU); Yoruba from Ibadan, Nigeria (YRI); Han Chinese from Beijing, China (CHB); and Japanese from Tokyo, Japan (JPT). Abbreviations within Finland: Southwest Finland (SWF); Satakunta (SAT); Häme (HAM); Southern Ostrobothnia (SOB); Swedish-speaking Ostrobothnia (SSOB); Savo (SAV); Northern Karelia (NKAR); Kainuu (KAI); Northern Ostrobothnia (NOB); Miscellaneous (MISC).

The aim of this study was to characterize the genetic variation of Finland, Sweden, Northern Germany and Great Britain together with the HapMap data (Fig. 1) on a finer level than previously possible, using 250,000 SNPs. In addition to analysing patterns of population differentiation, diversity and admixture in North Europe, we had a special interest on elucidating population structure within Finland. The populations of Central European background showed signs of only minor population differentiation, whereas the Swedes and Finns exhibited a stronger population structure–also within Finland–and decreased genetic diversity, both of which suggested a pronounced genetic drift among North Europeans.

## Results

### Analyses between populations

**Figure 2:**
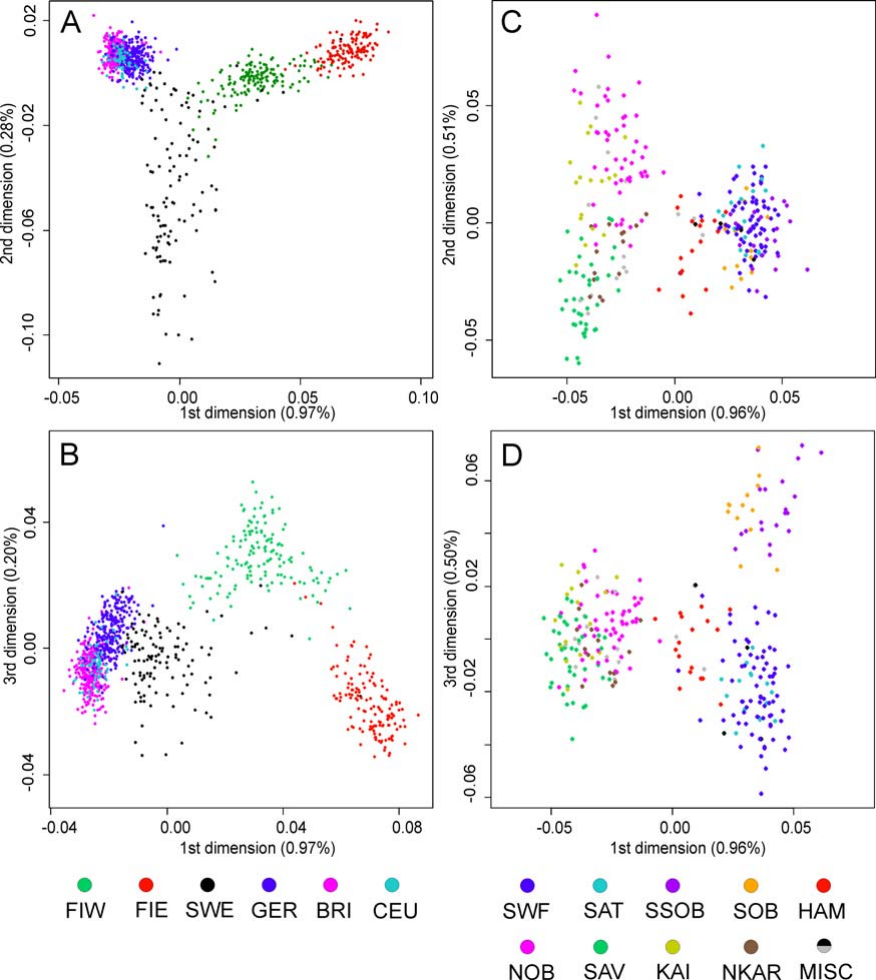
Multidimensional scaling plots of the identity by state matrices. Plots for the Europeans in the 1^st^ and 2^nd^ dimensions (a), and the 1^st^ and 3^rd^ dimensions (b), and the Finnish samples in the 1^st^ and 2^nd^ dimensions (c), and the 1^st^ and 3^rd^ dimensions (d). The label of each axis shows the proportion of the dimension’s eigenvalue to the sum of absolute eigenvalues of all dimensions. Abbreviations as in Figure 1. See also Figure S1 for three-dimensional animations.

**Figure 3:**
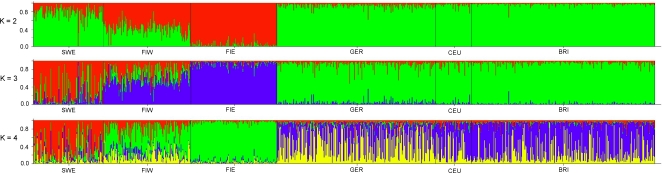
The Structure results for two, three and four clusters. Each individual is represented by a thin vertical line, and colours denote the clusters. Abbreviations as in Figure 1. The probabilities of the different clusterings are given in Supplementary Figure 2b.

After genotyping on Affymetrix 250K Sty SNP arrays (see Methods and Table S1 for success rates and quality criteria), the data from 1003 European individuals were first compared without prior population assignment in the analyses of pairwise identities by state (IBS) and calculations with the Structure software. In multidimensional scaling of the IBS distances, there were four clusters: Eastern Finns, Western Finns, Swedes, and a group including the Germans, British and CEU (from now on called ”Central Europeans„; Fig. 2a,b, Fig. S1a). The median IBSs between the European population pairs (Table 1), which are free of the potential bias caused by multidimensional scaling, indicated a closer relationship of Eastern v. Western Finns and Germans v. British, and largest differences between the Eastern Finns v. British and Eastern Finns v. Germans (p<10^−14^ for all population pairs except between Sweden v. Western Finland, Germany and Great Britain). The Structure analysis (Fig. 3, Fig. S2a,b) found most support for three or four clusters, one dominated by the Eastern Finns, one by the Swedes, and one by the Central Europeans; increasing the number of clusters did not bring out further differences. When only the Finnish samples were analysed with Structure, they formed two clusters (Fig. S2c), consisting of the Eastern and Western Finns, with only 1.8% of the samples associating more strongly to the cluster not corresponding to their geographic origin (data not shown). A Structure analysis of the three Central European populations combined found only one cluster.

**Table 1:** Pairwise F_ST_’s (lower diagonal) and the median IBS (upper diagonal) between population pairs.

	SWE	FIW	FIE	GER	BRI
SWE		0.7997	0.7990	0.7997	0.7997
FIW	0.0030		0.8005	0.7994	0.7993
FIE	0.0072	0.0032		0.7985	0.7982
GER	0.0021	0.0033	0.0084		0.8002
BRI	0.0024	0.0042	0.0094	0.0005	

All F_ST_’s differ from zero (p<10^−3^), and their 95% confidence intervals are ±0.0005 or narrower. For the IBS, p<10^−14^ for all population pairs except between Sweden v. Western Finland, Germany and Great Britain.

**Figure 4:**
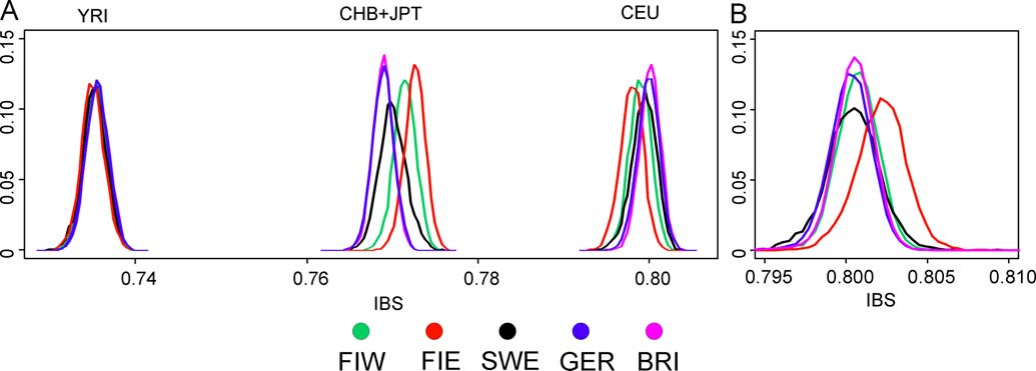
Distributions of pairwise identities by state. IBS between the five studied populations and each HapMap population (a) and within the populations (b). Within the four groups of comparisons, all distribution pairs differed significantly (p<4.6×10^−4^ for comparisons within the populations, p<10^−14^ with CEU and with CHB+JPT, and p<0.025 with YRI) except that in the comparisons with Asians, Germany and Great Britain did not differ. Abbreviations as in Figure 1.

When data from HapMap Han Chinese+Japanese and Yoruba individuals was included in the analysis, the MDS plot of IBS formed a triangle of the three continents in the first two dimensions, with the third dimension separating the European populations clinally from each other (Fig. S3). In the histograms of IBS between the five European populations and each HapMap population (Fig. 4a), the studied populations were most similar with the CEU and least similar with YRI. Interestingly, the similarity with the Asians varied between populations, being higher for Eastern Finns, Western Finns and Swedes than for the Germans and British (p<10^−14^ for all comparisons except for GER and BRI whose distributions did not differ). The same pattern was also observed when comparing the allele frequencies in the study populations and in CEU and CHB+JPT: the Eastern Finns had the largest proportion of SNPs deviating towards the Asian frequencies (Table S2; p<10^−5^), also when markers with smallest differences were excluded (data not shown).

**Figure 5:**
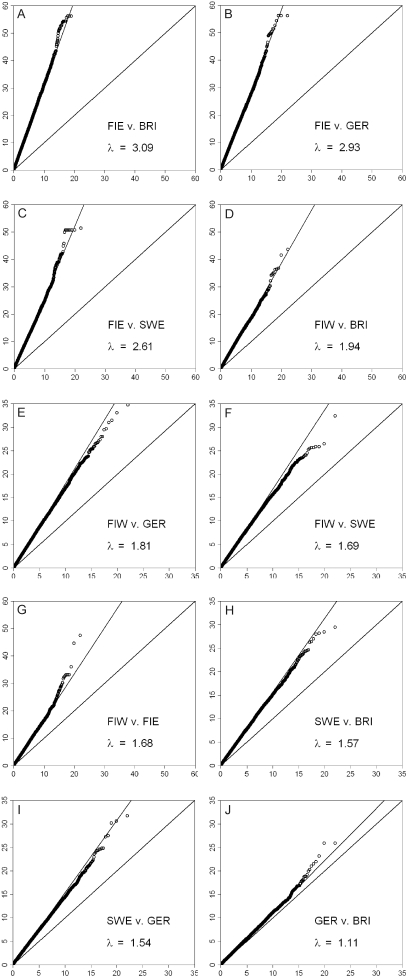
Quantile-quantile plots of allele frequencies between population pairs. λ denotes the overdispersion factor. One SNP with an observed value of ∼120 has been left out from all the plots with the Germans. Note the two different scales of the axes. Abbreviations as in Figure 1.

**Figure 6:**
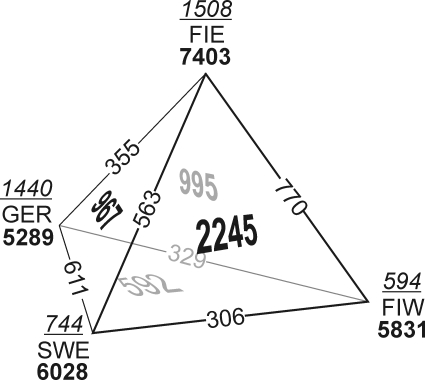
The number of monomorphic markers. The total number of monomorphic markers within each population is given in bold, and the markers that are monomorphic exclusively in one population are in underlined italics. The edges of the tetrahedron denote the markers that are monomorphic only in two populations, and the faces correspond to monomorphy shared between three populations. 21 319 SNPs that were monomorphic in all the four populations are not included in the figure. Abbreviations as in Figure 1.

Quantile-quantile plots of pairwise allele frequency differences (Fig. 5) and F_ST_ calculations (Table 1) showed a pattern of the largest differences being between Eastern Finland versus Great Britain, Germany and Sweden (F_ST_ = 0.0072–0.0094) and the smallest between the British and Germans (F_ST_ = 0.0005). All the FSTs differed from zero (p<10^−3^), and most of them also differed from each other (the range of 95% confidence intervals ±0.0005 or less). The F_ST_ over all populations was 0.0040 (p<10^−4^). Notably, there was no indication of the closer relationship of the two Finnish populations that was observed in the IBS analysis of individuals (Fig. S4a). The relationships between populations could also be measured by the number of shared monomorphic markers in Finland, Sweden and Germany (Fig. 6). There, the total number of monomorphic and uniquely monomorphic markers were highest in Eastern Finland, pairwise sharing was highest between Eastern and Western Finland, and three-way sharing between the two Finnish populations and Swedes. A total of 19088 markers were monomorphic in all four populations and an additional 2231 when the populations were sampled to equal size, and these were excluded from the analysis.

### Variation within populations

**Figure 7:**
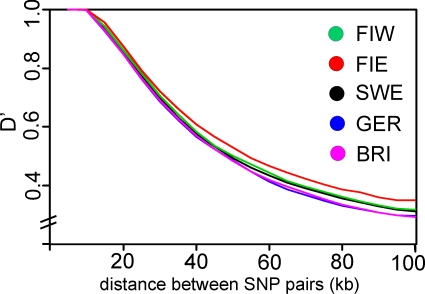
Linkage disequilibrium as a function of distance between marker pairs. Median D' in overlapping 10 kb windows at 5 kb intervals is plotted for each population. All differences were significant (p<6.2×10^−10^), except between Germany and Great Britain. Abbreviations as in Figure 1.

The IBS between individuals within populations (Fig. 4b) was highest for Eastern Finland and lowest in Germany (p<4.6×10^−4^). Differences in the extent of linkage disequilibrium were highly significant (p<6.2×10^−10^) for all population pairs except Germans and British (Fig. 7): LD was highest in Eastern Finns and lowest in Germans and British. Marker and sample heterozygosities, inbreeding coefficients and minor allele frequency distributions had only very small, although mostly significant, differences between the populations (Table S3). When the European populations were analysed separately in Structure, none showed evidence of a substructure.

The information about the grandparental birthplaces of the Finnish samples enabled a more detailed analysis of population structure within Finland. In the multidimensional scaling plot of IBS within Finland (Fig. 2c,d, Fig. S1b), the first dimension showed the division to Eastern and Western Finland; the Häme samples settled between the clusters. The second dimension showed a north-south gradient within Eastern and the third dimension within Western Finland. Here the Swedish-speaking Ostrobothnians showed no separation from their Finnish-speaking neighbours, whereas in the MDS plot of the European populations, the Finnish samples closest to the Swedes were almost exclusively Swedish-speakers (data not shown), and in the Structure analysis the Swedish-speaking Finns showed twice as large an admixture with the Sweden-dominated cluster as the other Western Finnish samples did (48.9% versus 24.6%, data not shown). In the analysis of isolation by distance (Fig. S5), the correlation of genetic and geographic distances between pairs of Finnish individuals was 0.31 (p<10^−6^).

## Discussion

Analysing large numbers of autosomal markers has advantages over the traditional tools of population genetic studies. Mitochondrial DNA and Y-chromosomal markers represent only two loci and thus do not fully capture the evolutionary history throughout the whole genome, and limited numbers of autosomal loci may lack the power to detect differences especially between closely related populations. In this study, we used 250,000 SNPs to elucidate the population structure and differentiation in Northern Europe by analyzing carefully ascertained samples from Eastern and Western Finland, Sweden, Germany and Great Britain. Our results revealed a relatively strong population structure within Finland, and a small but significant differentiation between all the populations, although especially the Germans and British appeared genetically very homogeneous.

The F_ST_ values showed a pattern of very small yet statistically significant differences between the populations. The overall F_ST_ (0.0040) was equal to the F_ST_ between European regions calculated from a similar set of markers [9]. The population structure among Eastern and Western Finland (F_ST_ = 0.0032) was similar to that between the Icelandic subpopulations (0.0034) [38], but much stronger than what has been observed between Northern and Southern Germany (0.00017) [35], and stronger than between some of the countries in our data, despite the shorter geographic distance. A comparable structure within Finland has been observed earlier with Y-chromosomal and autosomal markers [23], [27]. The differences between populations detected with F_ST_ and other measures accounted for such a small proportion of the total genetic variation that large numbers of SNPs are needed to observe them, once again illustrating how most of the human genetic variation is found between individuals instead of populations [39]. Even small differences between populations can be interesting regarding population history, but elucidating their phenotypic significance will require further studies.

The MDS plot of the European populations showed a pattern of population differences that was consistent with our other analyses and earlier observations of a greater degree of differentiation in the geographical extremes of Europe [3], [5], [7], [9]–[11]. Our German, British and CEU samples formed a single cluster, possibly due to a lack of neighbouring reference populations, and contrary to studies with a more comprehensive sampling from Central Europe [7], [9]. The Swedes showed a wider spreading than the other populations, but this was supported neither by diversity calculations nor by a more detailed comparison of the IBS and MDS distance matrices (results not shown). Thus, the differential spread was at least partly an artefact of the MDS, where the representation in a few dimensions likely fails to capture all aspects of complex data. Thus, as visually attractive as the MDS plots are, they must be interpreted with caution and, if sample sizes allow, be accompanied with analyses based on allele frequencies.

The MDS analysis of Finns showed a pattern resembling their geographic origins, although with some overlap of the provinces. A similar regional clustering of individuals has been seen in the Swiss [9], but not in Great Britain [4]. The increased Swedish contribution among the Swedish-speaking Finns agrees with earlier findings [27], [40], as well as with their medieval Swedish origin [14]. Interestingly, in the MDS plots the Finnish-Swedes stood out from the rest of Western Finland only when Sweden was included in the analysis, which highlights the importance of relevant reference populations also when detecting patterns of variation within a country.

The extreme features of Eastern Finland-high linkage disequilibrium, high similarity within the population, increased number of monomorphic markers and divergence from the other populations-are in accordance with earlier studies [20]–[30]. They are likely caused by population history: the young age of the population, founder and bottleneck effects, and substantial genetic drift attributable to small population size. The settlement of Eastern Finland from the province of South Savo beginning in the 16th century led to serial founder effects, and genetic drift remained strong in the small and isolated breeding units during the following centuries [17], [18]. These local processes were also reflected in the regional MDS clustering of individuals within Eastern and Western Finland. Similar processes, although much less extreme in magnitude, have probably caused the slight decrease in diversity observed in Sweden and Western Finland. Conversely, the Germans and British showed much less divergence, and their LD was significantly lower and diversity higher than among the Nordic populations.

Another factor behind the outlier status of Finland could be admixture with other populations outside the studied region. Indeed, the comparison to the Asian HapMap samples revealed interesting differences between the studied populations, with the Nordic populations and especially Eastern Finns appearing to harbour a significantly stronger Asian affinity than Central Europeans. A similar eastern influence has been observed in Y-chromosomal, mitochondrial DNA and autosomal studies of the Finns [5], [20]–[23], consistently with archaeological and linguistic data. A small degree of Saami admixture has been observed among the Finns [41] and could also contribute to the differentiation observed in this study, but it could not be detected in the absence of reference data. Thus, the possible eastern contribution observed among the Finns supports the earlier studies done with a more limited number of markers, although a full synthesis of past migration waves is beyond the scope of this study and would require additional data.

In this study, the potential bias caused by limited sample size should not be a major problem, since the sample sizes were similar or larger than those commonly used in population genetic studies. Another putative source of error, genotyping centre artefacts between datasets, is difficult to exclude completely. However, the data for Finland and Sweden comes from a single genotyping centre, and thus analyses within the dataset are free from this potential bias. The genetic differences between the German and British datasets are small (F_ST_ = 0.0005, λ = 1.11) despite being genotyped in different laboratories, and thus these datasets seem comparable. Additionally, the bias in SNP ascertainment for the chips and in the LD-based formation of smaller datasets (Table S1) may affect the sensitivity of the markers to detect population structure, and thus the exact values of e.g. F_ST_ [42]. A further important factor in population genetic research is the geographical scale of sampling. Indeed, our German sample is from a region with considerable Scandinavian admixture [37]. Consequently, the German sample presumably captures neither the full extent of diversity and variability within Germany nor unbiased relationships between the whole populations. Within Finland, the observed sharp genetic borderline is probably partly explained by the gap between Western and Eastern Finland in our sampling, and a geographically continuous sampling could have yielded a more clinal pattern of genetic variation. Nonetheless, the extent of the differences between the areas now sampled would obviously not change.

In the analysis of differences between populations, the patterns observed in individual-based analysis and in calculations based on allele frequencies usually correlated well. However, in the IBS analysis the Eastern and Western Finns appeared relatively closer to each other than in the quantile-quantile plots or F_ST_ (Fig. S4a). Figures S4b,c show the expected values of mean markerwise IBS and chi-square test statistic for all combinations of allele frequencies in two populations, and demonstrate that the measures behave differently with respect to allele frequencies. This difference explains why two population pairs could show disparate distances with one measure and similar with the other. The measures could also vary in their sensitivity to various patterns of allele frequency differences and thus to the population genetic processes that have caused the patterns.

Population isolates are easily considered homogeneous without further evaluation. Many of the advantages of using population isolates in gene mapping [15], [43] are a consequence of factors that also make the population subunits vulnerable to genetic drift and may lead to population stratification. Our results show that these factors have had a substantial effect in the patterns of genetic variation in Northern Europe, where the populations show a greater degree of differentiation than the more stable and admixed Central European populations. Because the detected structure within the Finnish population is of the magnitude that has been suggested to be a potential source of bias in association studies [1], [2], [38], our results suggest that attention to population substructure may be needed to ensure the quality of association studies that are performed using Finnish samples. In fact, the differences between Eastern and Western Finns were of the same magnitude as the differences between Swedes and British, and much stronger than those between British and Germans. Thus, relevant units of genetic variation often do not correspond to preconceived political, linguistic or even cultural borders.

## Materials and Methods

We genotyped 139 genomic DNA samples from Eastern Finland, 141 samples from Western Finland and 113 samples from eastern Sweden with the Affymetrix 250K Sty SNP array (Santa Clara, CA) (Fig. 1). All the sample donors were males. The geographical origin of the Finnish samples was assessed according to grandparental birthplace, but no detailed ancestry information was available for the Swedes. Additionally, we used data for 256 male control samples from the PopGen cohort from Kiel area in Schleswig-Holstein in Northern Germany [44]. All the samples were collected with informed consent according to the principles of the Declaration of Helsinki, and the project was approved by the ethics committees of the Finnish Red Cross, Umeå University, and the Kiel Medical Faculty. We also used data from 296 male controls of the 1958 birth cohort kindly provided by the Wellcome Trust Case Control Consortium [4] and sampled according to the region information to cover the entire Great Britain. Furthermore, we obtained 250K Sty array genotypes of the unrelated HapMap [12] individuals from Affymetrix: 58 Utah residents with ancestry from northern and western Europe (CEU), 57 Yoruba from Ibadan, Nigeria (YRI), 42 Japanese from Tokyo, Japan (JPT) and 45 Han Chinese from Beijing, China (CHB).

The genotype calling was done by the BRLMM algorithm in the Affymetrix GeneChip Genotyping Analysis Software (GTYPE) version 4.1, and the quality control procedures followed for the most part the Wellcome Trust Case Control Consortium standards [4] (Table S1). Samples with success rate below 97% were excluded. For markers, the exclusion limits were 95% for success rate, p<0.001 for deviation from Hardy-Weinberg equilibrium in any of the populations, and 0.005 for minor allele frequency. This yielded a total of 201 011 SNPs and 1147 samples that passed the quality control. Additionally, two smaller marker sets were constructed by LD-based SNP pruning: 68469 SNPs with r^2^<0.2, and 6369 SNPs with minor allele frequency >0.1 and r^2^<0.02. The former set was used for the IBS and inbreeding analyses and the latter for Structure and F_ST_ analyses. Many of the analyses were performed without the HapMap populations in order to avoid extensive sampling or possible bias due to their lower sample sizes. We performed most of the analyses in parallel in Plink version 1.00 (http://pngu.mgh.harvard.edu/purcell/plink/) [45] and the R 2.6.2 (www.R-project.org) [46] package GenABEL 1.3–5 [47] to eliminate human and software errors.

We calculated pairwise identities by state (IBS) for all samples, and performed classical multidimensional scaling (MDS) on the identity matrices for the total data and for the European and Finnish datasets separately. The informativeness of the presented dimensions was assessed by calculating the proportion of their respective eigenvalues to the sum of absolute eigenvalues. Distributions of IBS in sample pairs within and between populations, as well as marker and sample heterozygosities and inbreeding coefficients were calculated in GenABEL, together with distributions of minor allele frequencies in the populations. Geographic coordinates for each Finnish individual were determined as the mean of grandparental birthplace coordinates, and the geographic distances between all the individuals were calculated as great-circle distances in R package fields [48]. The correlation between the geographic and genetic distances (1-IBS) was measured by Mantel test as implemented in R package ade4 [49]. We estimated the extent of linkage disequilibrium (LD) in each population by calculating D' between all marker pairs within 100 SNPs from each other, using for each marker pair the median result of the values based on the frequency estimates of all four haplotypes calculated with the E-M algorithm in Plink. Population structure was assessed also by Structure 2.2 software [50] with the admixture model and 10000 burn-ins and iterations, doing four separate runs for each K. Estimation of the correct K was based on visual inspection of the respective probabilities and of the distribution of the populations among the inferred clusters. No substructure was inferred when the probability was largest for K = 1. For F_ST_ calculations we used Arlequin 3.11 [51]; the p-values and 95% confidence intervals are based on 10100 permutations. The allele frequency differences in population pairs were tested with markerwise 1-df chi-square tests in Plink, and the deviation from expected chi-square distribution was visualized in quantile-quantile plots. Their overdispersion factor (λ) was calculated as a ratio of the means of the lowest 90% of the observed and expected chi-square values as in [52]. Additionally, we calculated the number and distribution of markers that were monomorphic in at least one of the populations; this analysis was performed only for the Finns, Swedes and Germans due to the difficulty of visualising multiple population comparisons.

To study the extent of eastern influence, we counted in each of the five European populations the number of markers where the population's allele frequency and the CHB+JPT allele frequency deviated from the CEU allele frequency to the same direction, and the number of markers where the allele frequencies deviated in opposite directions. We then compared the numbers to the null hypothesis that all the five populations stem from the same proto-European population (approximated by the CEU frequencies) from which they have subsequently diverged via genetic drift in the absence of admixture. In such a case, one would expect the number of markers drifting into a given direction (e.g. towards the Asian frequencies) to be similar across the populations, whereas a varying degree of eastern admixture in each population would result in disparate marker proportions. Using the number of deviating markers instead of the absolute size of the deviations should even out some of the effects of differing extent of drift in the populations.

The statistical significancies of the differences between the distributions of each analysis were tested in R by first assessing their normality by a Shapiro-Wilk test. As all were strongly non-normal, the pairwise analyses (LD, marker heterozygosities) were done with a sign test; in the independent analyses (allele frequencies, sample heterozygosities, IBS distributions, inbreeding coefficients), an overall significance of the difference was first calculated from a Kruskal-Wallis one-way analysis of variance, and if that was significant, the differences were further located by pairwise comparisons with a Mann-Whitney U test. The medians given in Tables 1 and S3 are calculated from the datasets listed in Table S1, but to avoid possible effects of sample size, the significance testing of marker heterozygosities, inbreeding and allele frequencies was done on populations sampled to n = 113. The statistical significance of differences in the number of SNPs whose frequencies deviated towards or away from the Asian frequencies was assessed by a 2×5 chi-square test. A Bonferroni correction has been applied to the reported significance levels to correct for the number of pairwise comparisons within each analysis.

## Supporting Information

Table S1 Quality control parameters and the different datasets used in analyses
(0.04 MB XLS)

Table S2 The number of SNPs per population that have a frequency deviation from CEU to the same or opposite direction as Asia (CHB+JPT). The markers with identical frequencies in either CEU and the studied population or CEU and CHB+JPT have been excluded. The proportions differ significantly (p<10-5).
(0.02 MB XLS)

Table S3 Summary table of population statistics
(0.02 MB XLS)

Figure S1 Animation of the three-dimensional multidimensional scaling plot of the identity by state matrix of the Europeans (a), and the Finnish samples (b), with the legend in (c). The file can be opened e.g. in most internet browsers. Abbreviations as in Figure 1.
(20.95 MB GIF)

Figure S2 Admixture proportions of the European individuals in a Structure analysis of K = 3 (a); and the probabilities of different numbers of clusters in the Structure analysis of the European dataset (b), and the Finnish dataset (c).
(0.54 MB TIF)

Figure S3 Multidimensional scaling plots of the identity by state matrices for the whole dataset. Plots in the 1st and 2nd dimensions (a), and the 1st and 3rd dimensions (b). The label of each axis shows the proportion of the dimension's eigenvalue to the sum of absolute eigenvalues of all the dimensions. Abbreviations as in Figure 1.
(0.50 MB TIF)

Figure S4 Median IBS and overdispersion factor (lambda) of the quantile-quantile plot for each population pair (a), and values of chi-square test statistic (b) and expected mean IBS (c) for combinations of allele frequencies in two populations. In the chi-square calculation, samples from both populations are assumed to be size n; the actual test statistic will be n times the plotted value. The IBS calculation assumes Hardy-Weinberg equilibrium. Obviously, the IBS is highest (difference smallest) in a marker whose allele frequency is either high or low in both populations, whereas the chi-square value is less dependent on the actual size of the allele frequencies and more directly related to their difference. Thus, a given set of genome-wide allele frequencies can lead to different results in different analyses. Note that low minor allele frequencies are most common in Eastern Finland.
(0.71 MB TIF)

Figure S5 Geographic versus genetic distance for all Finnish individual pairs. The p-value is based on 10 000 replications. Correlation coefficient is 0.31 (p<10-6).
(0.15 MB TIF)
