# Supplementary figures and images for: Genome-Wide Analysis of Single Nucleotide Polymorphisms Uncovers Population Structure in Northern Europe
Source: PLoS One. 2008 Oct 24;3(10):e3519. doi: 10.1371/journal.pone.0003519 (PMC2567036; doi:10.1371/journal.pone.0003519)

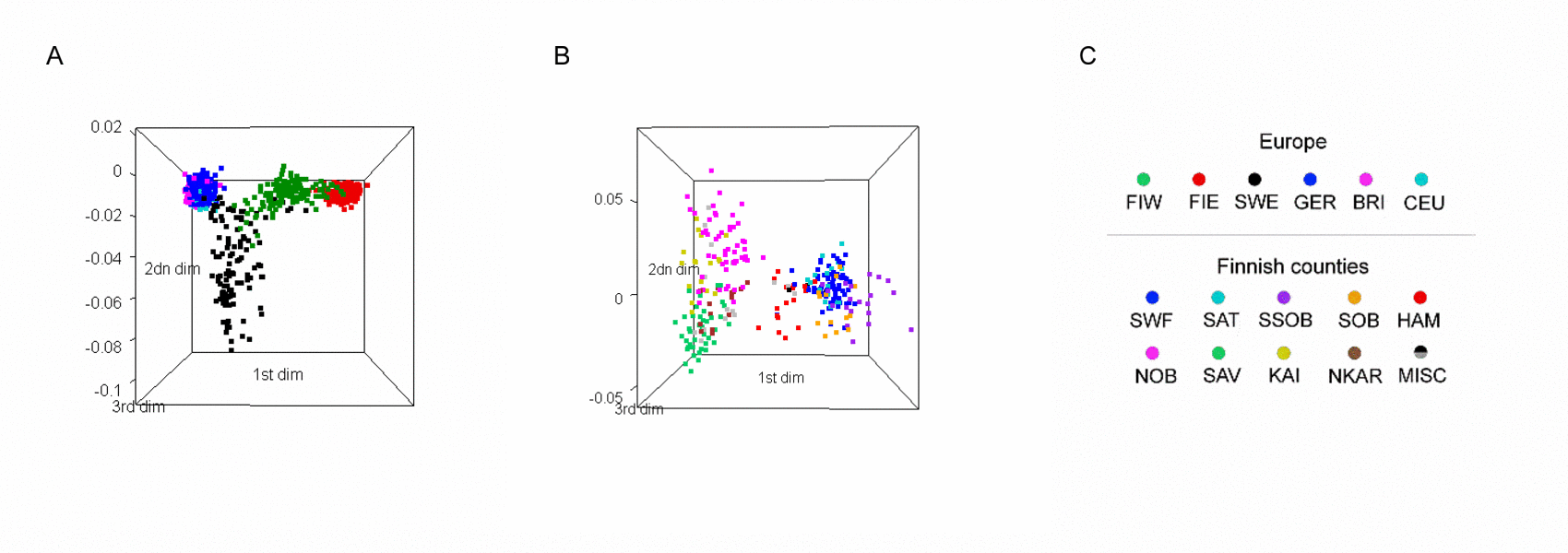

Supplement: Figure S1 — Animation of the three-dimensional multidimensional scaling plot of the identity by state matrix of the Europeans (a), and the Finnish samples (b), with the legend in (c). The file can be opened e.g. in most internet browsers. Abbreviations as in Figure 1. (20.95 MB GIF) [file pone.0003519.s004.gif]

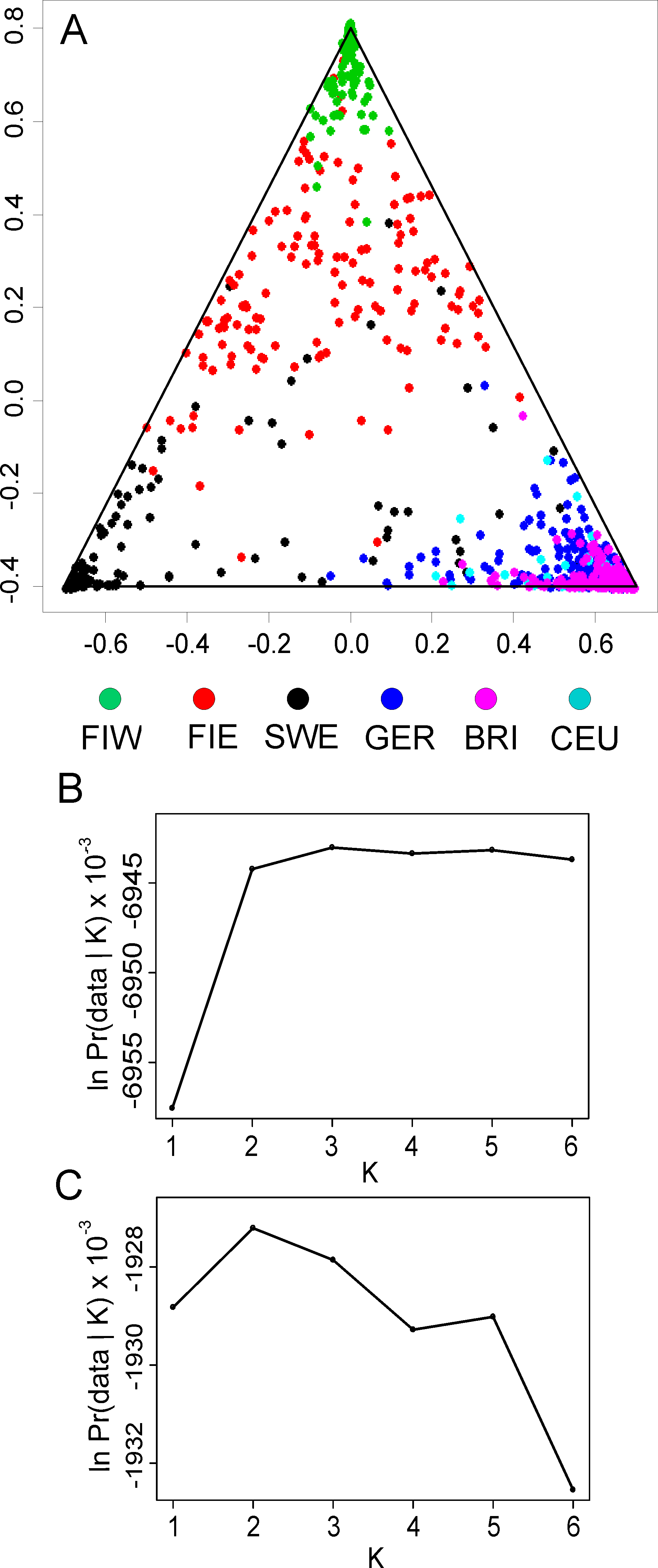

Supplement: Figure S2 — Admixture proportions of the European individuals in a Structure analysis of K = 3 (a); and the probabilities of different numbers of clusters in the Structure analysis of the European dataset (b), and the Finnish dataset (c). (0.54 MB TIF) [file pone.0003519.s005.tif]

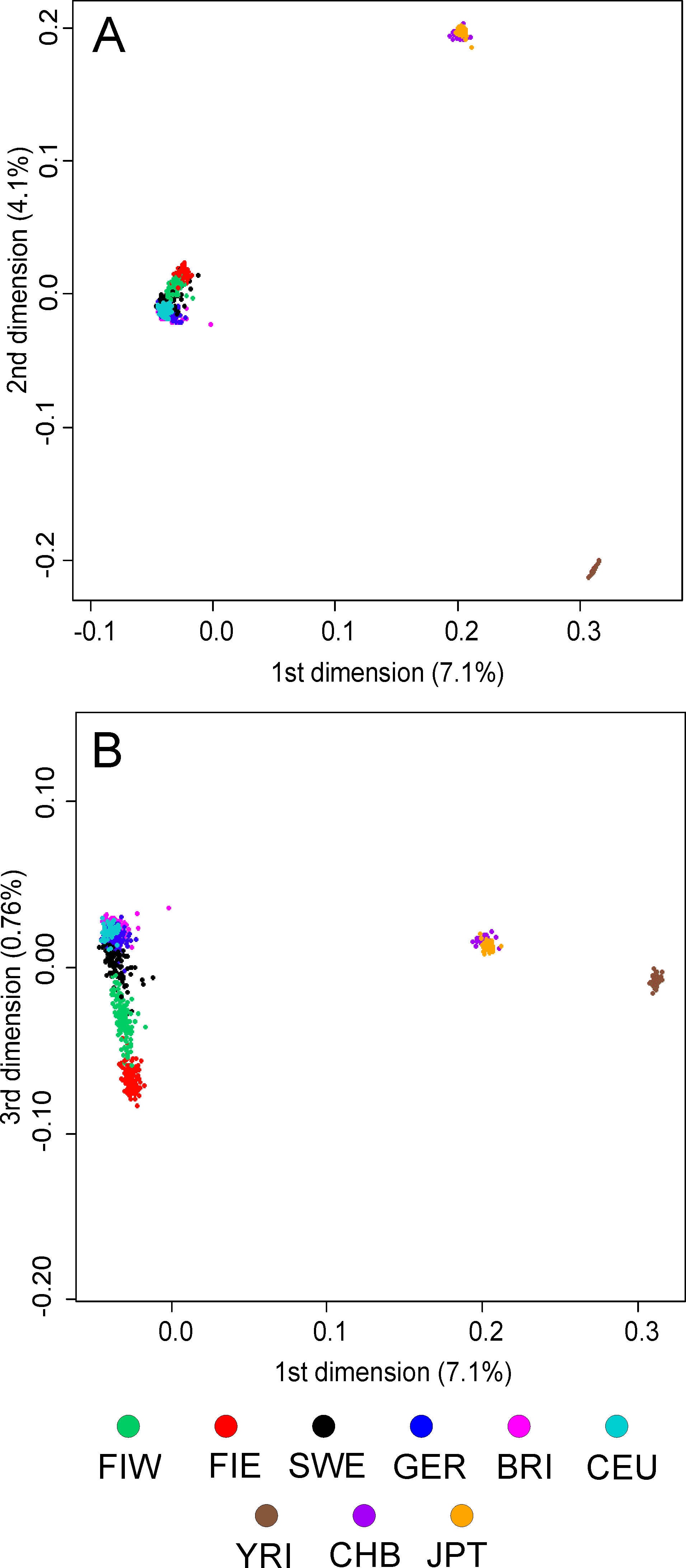

Supplement: Figure S3 — Multidimensional scaling plots of the identity by state matrices for the whole dataset. Plots in the 1st and 2nd dimensions (a), and the 1st and 3rd dimensions (b). The label of each axis shows the proportion of the dimension's eigenvalue to the sum of absolute eigenvalues of all the dimensions. Abbreviations as in Figure 1. (0.50 MB TIF) [file pone.0003519.s006.tif]

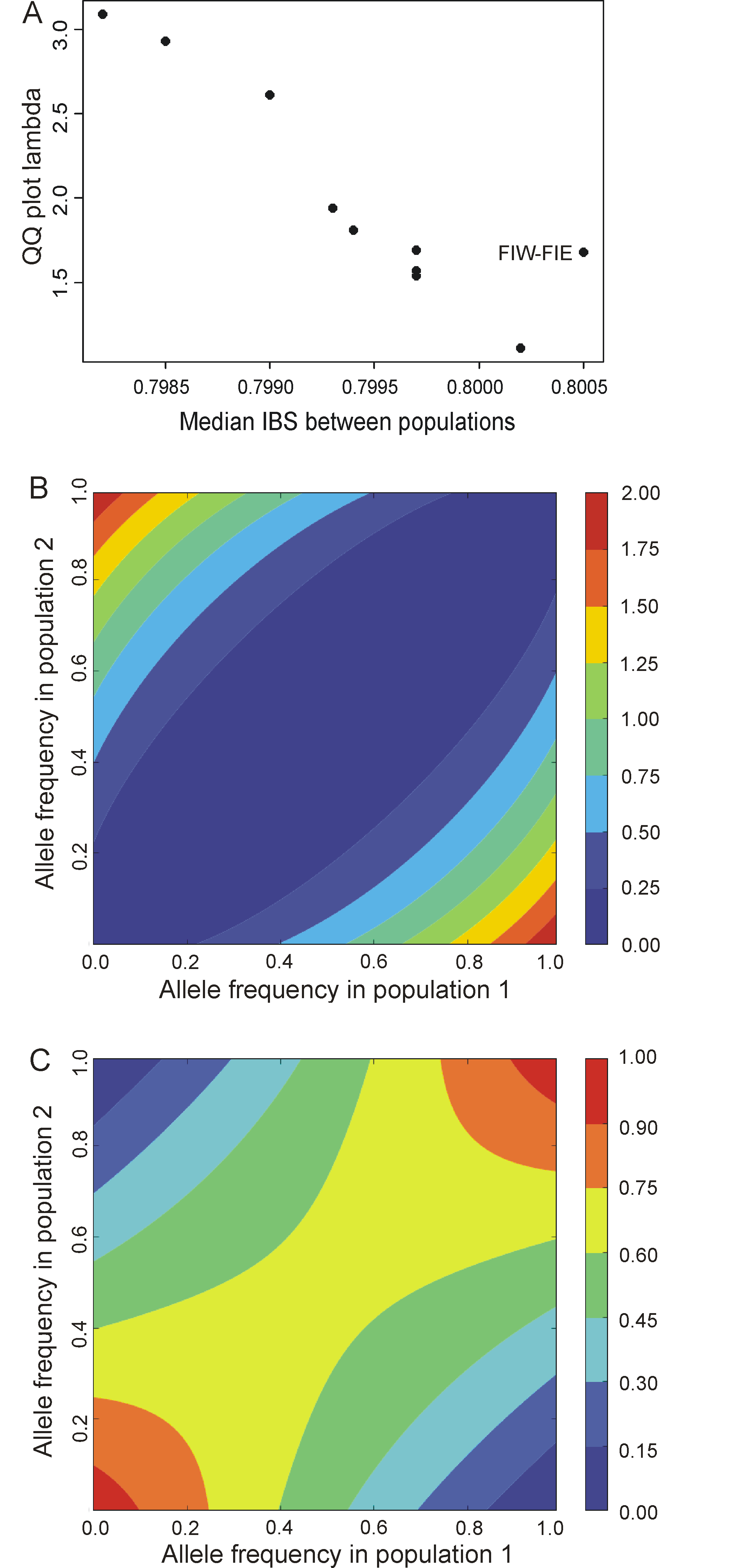

Supplement: Figure S4 — Median IBS and overdispersion factor (lambda) of the quantile-quantile plot for each population pair (a), and values of chi-square test statistic (b) and expected mean IBS (c) for combinations of allele frequencies in two populations. In the chi-square calculation, samples from both populations are assumed to be size n; the actual test statistic will be n times the plotted value. The IBS calculation assumes Hardy-Weinberg equilibrium. Obviously, the IBS is highest (difference smallest) in a marker whose allele frequency is either high or low in both populations, whereas the chi-square value is less dependent on the actual size of the allele frequencies and more directly related to their difference. Thus, a given set of genome-wide allele frequencies can lead to different results in different analyses. Note that low minor allele frequencies are most common in Eastern Finland. (0.71 MB TIF) [file pone.0003519.s007.tif]

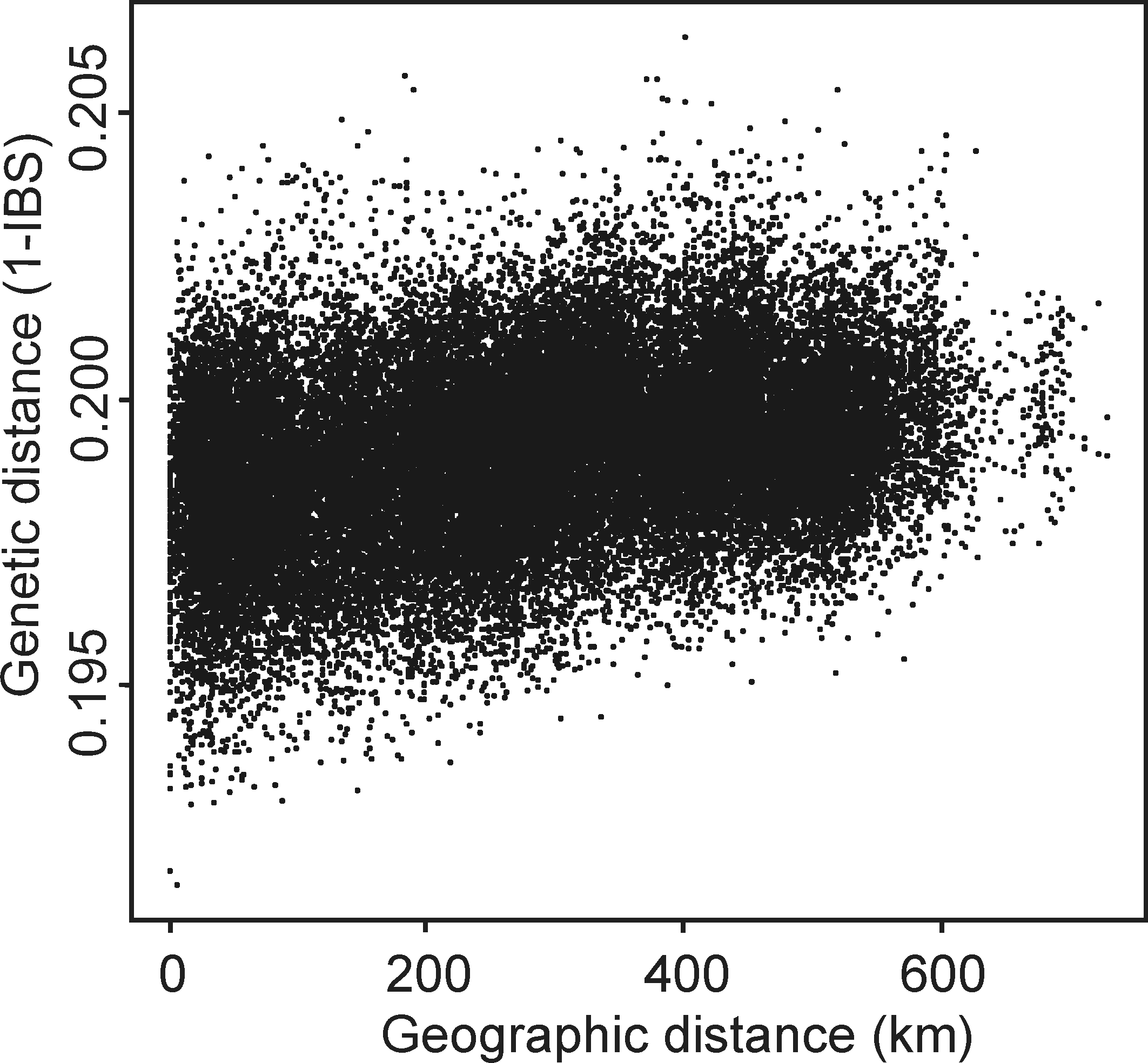

Supplement: Figure S5 — Geographic versus genetic distance for all Finnish individual pairs. The p-value is based on 10 000 replications. Correlation coefficient is 0.31 (p<10-6). (0.15 MB TIF) [file pone.0003519.s008.tif]
